# Variation in cancer risk between organs can not be explained by the degree of somatic clonal expansion

**DOI:** 10.1007/s44307-024-00025-9

**Published:** 2024-05-23

**Authors:** Di Zhang, Ao Zhang, Xionglei He, Shanjun Deng

**Affiliations:** State Key Labratory of Biocontrol, School of Life Sciences, Sun Yat-San University, Guangzhou, 510275 China

**Keywords:** Somatic mutations, Allele frequency spectrum, Clonal expansion, Lifetime cancer risk

## Abstract

Somatic clonal expansion refers to the proliferation and expansion of a cell clone within a multicellular organism. Since cancer also results from the uncontrolled proliferation of few cell clones, it is generally believed that aging-associated somatic clonal expansion observed in normal tissues represents a precancerous condition. For instance, hematological malignancy is often preceded by clonal hematopoiesis. However, the precise connection between cancer and somatic clonal expansion remains elusive in solid organs. In this study, we utilized a straightforward method to assess the relative quantitative degrees of clonal expansion in nine human organs. Our findings reveal that the degree of clonal expansion varies across different organs while remaining consistent among different individuals. Contrary to the general belief, we did not identify any significant correlation between lifetime cancer risk and the degree of lifetime somatic clonal expansion. For example, the lifetime risk of colorectal cancer is approximately 20 times higher than that of esophageal cancer, yet the former exhibited the lower degree of clonal expansion than the latter. Our results suggest that somatic clonal expansion represents an evolutionary process distinct from carcinogenesis in normal tissues, providing novel perspectives on precancerous conditions.

**Supplementary Information:**

The online version contains supplementary material available at 10.1007/s44307-024-00025-9.

## Introduction

Cancer development is characterized by the abnormal proliferation of somatic cells. The process is driven by genetic alterations, especially in oncogenes or tumor suppressor genes (Armitage and Doll 1954; Fearon and Vogelstein 1990; Fisher 1958; Knudson 1971; Moolgavkar and Knudson 1981; Nordling 1953). To investigate how clonal expansion leads to tumor formation, a paradigmatic framework has been proposed in colorectal tumorigenesis. With the sequential acquisition of mutations in APC, KRAS, TP53, and SMAD4 over time, advantageous clones expand from normal epithelial cells to metastatic cancer (Fearon and Vogelstein 1990; Vogelstein et al. 2013), indicating the inseparable relationship between accumulated mutations of tumor cells and the latent evolutionary process during tumorigenesis.

Recently, plenty of studies have shed light on the prevalence of somatic clonal expansion through deep DNA sequencing. Various organs were investigated in healthy individuals, including blood (Fabre et al. 2022; Lee-Six et al. 2018; E. Mitchell et al. 2022; S. R. Mitchell et al. 2021; Xie et al. 2014), skin (Martincorena et al. 2015), esophagus (Martincorena et al. 2018; Yokoyama et al. 2019), bronchus (Yoshida et al. 2020), liver (Brunner et al. 2019), endometrium (Moore et al. 2021; Suda et al. 2018), and bladder (Lawson et al. 2020). Notably, almost 90% of genes responsible for driving somatic clonal expansion in normal tissues are shared with genes associated with tumor development in multiple organs (Acha-Sagredo et al. 2022). The presence of common driver mutations strongly implies that clonal expansion in normal tissues is the precursor stage of tumorigenesis. Based on the explicit relationship between clonal expansion and tumorigenesis, the degree of clonal expansion has been widely used in medical diagnosis, particularly in the hematopoietic system. For instance, individuals with clonal hematopoiesis-related mutations were found to face a roughly tenfold higher risk of developing myeloid neoplasms compared to those without such mutations (S. R. Mitchell et al. 2021). In addition, approximately 1% of individuals with clonal hematopoiesis develop leukemia annually, highlighting the clonal hematopoiesis as a precursor to hematological malignancies and its clinical diagnostic utility (Xie et al. 2014; McKerrell et al. 2015; Chan et al. 2020).

Although the intense relationship between somatic clonal expansion and tumorigenesis found in the hematopoietic system has been well investigated in the past few years, it remains an open question in solid organs. The gap is primarily due to the lack of sufficient data for measuring somatic clonal expansion across multiple organs in the population. Recently, Li et al. conducted a comprehensive analysis on 1,737 tissue microbiopsies collected from nine organs in five elderly individuals without known health issues. The study revealed widespread clonal expansions in normal organs (2021). It provides an excellent opportunity to directly investigate the relationship between somatic clonal expansion and cancer risk in solid tissues.

In this study, we proposed a straightforward method to quantify the degree of somatic clonal expansion and identified a strong consistency in clonal expansion across multiple organs among individuals. Then, we conducted an exploration of the relationship between the degree of clonal expansion and lifetime cancer incidence.

## Method

### Assessing the reliability of MVAF for measuring the degree of clonal expansion

The comparative dataset contrasting samples from normal and cirrhotic livers was sourced from a prior investigation conducted by Brunner et al (2019). Their study employed a slice-and-sequence analysis approach on liver samples obtained from a diverse group of individuals. This research notably underscored a substantial divergence in clonal expansion patterns between livers in a normal state and those afflicted by inflammation and cirrhosis. We considered using the data of SNPs confirmed by the authors (Brunner et al. 2019). The MVAF values were calculated by taking the mean variant allele frequency across all SNPs for each biopsy. Then, the degree of clonal expansion was calculated by taking the mean MVAF across all microbiopsies from that liver. The significant differences in the degree of clonal expansion in different states were compared using the Mann–Whitney Wilcoxon test.

### Applying MVAF to assess the degree of clonal expansion

Comprehensive somatic mutation landscape data for each organ across different donors was acquired from the research published by Li et al. (2021). This dataset encompasses somatic mutation frequency data derived from whole-exome sequencing of nine normal solid organs across five individuals. In subsequent analyses, we used the data of 53,875 SNPs confirmed by the authors. Approximately 34–50 biopsies were collected from each tissue using laser capture microdissection (LCM) (Supplementary Fig. 1). It’s noteworthy that, with the exception of the pancreas and liver, which were sampled from normal parenchyma, all other tissues were obtained from normal epithelial regions.

Similar to the prior analysis, the MVAF values were calculated by taking the mean variant allele frequency across all SNPs for each biopsy. Then the degree of clonal expansion for each organ in every individual was obtained by taking the mean MVAF across all biopsies of that organ. Type II ANOVA was employed to simultaneously analyze the contributions of both the organ factor and the donor factor for the variation in mean MVAF. The contribution proportion is calculated as the ratio of the variance of the component divided by the total variance.

For investigate the clonal expansion degree in blood sample, we downloaded the dataset from Mitchell et al. (2022). We selected the data from three young adult donors (KX001, KX002, AX001) and two old donors (KX008, KX004) who were healthy. Then we calculated the mutations frequency within all samples and summarized with mean VAF to evaluate the degree of clonal expansion of each individual.

### Correlation analysis for the degree of clonal expansion and cancer risk

Lifetime cancer risk data was downloaded from the study published by Tomasetti et al. (2015). This comprehensive study collected and processed cancer risk dataset for multiple organs, primarily sourced from the Surveillance, Epidemiology, and End Results Program (SEER) database (http://seer.cancer.gov/statfacts/), and convincingly established a robust correlation between cancer risk and the number of divisions of stem cells in that tissue. For our analysis, we exclusively selected cancer risk data corresponding to the organs sampled using LCM. To further substantiate our findings, we accessed cumulative cancer incidence data for the Chinese population from the Global Cancer Observatory (GCO, https://gco.iarc.fr/) provided by the WHO. The data based on the GCO estimates of incidence for the year 2020 in 185 countries including China, is presented as rates per 100,000 population and has been age-standardized.

We calculated the average degree of clonal expansion for each organ across the five elderly populations. Then we used the Spearman rank correlation coefficient and the Pearson correlation coefficient to evaluate the relationship between the average degree of clonal expansion and lifetime cancer risk among the human population. The list of 147 somatic driver genes was downloaded from the review published by (Acha-Sagredo et al. 2022). The list of 738 tumor-related driver genes was obtained from the Cancer Gene Census (CGC) in the Catalogue Of Somatic Mutations In Cancer (COSMIC, https://cancer.sanger.ac.uk/cosmic)(Tate et al. 2019). To filter SNPs in somatic driver genes, cancer driver genes, or both types of genes separately, we used annotation files of GRCh37 version from the Ensembl genome browser to ensure consistency in gene positions. We calculated MVAF across filtered 1,766 SNPs in somatic driver genes, fliltered 4,334 SNPs in cancer driver genes, and total filtered 5,137 SNPs locating in both types of genes separately. We also categorized SNPs based on their positions, calculating the MVAF for SNPs located within exons, Coding DNA Sequences (CDS), and Untranslated Regions (UTR). In the end, we opted to calculate VAF for each biopsy using alternative metrics such as the median, quantile 90, and maximum of VAF instead of MVAF to indicate the degree of clonal expansion. The analysis is consistent by taking average to represent the degree in organ-level and the population-level. Subsequent correlation relationships were analyzed using the same method as described above.

## Result

### Quantitative analysis in the degree of clonal expansion using Mean Variant Allele Frequency (MVAF)

Given that the somatic mutation frequency can substantially increase during the clonal expansion process within a biopsy, the mean variant allele frequency (MVAF) within a biopsy can serve as a straightforward indicator reflecting the degree of clonal expansion. Liver is an example organ for researching the clonal expansion because of its regeneration capacity, and the degree of clonal expansion in liver cirrhosis is higher in than normal tissues due to the cell division events are more active in injured livers. Brunner et al. collected a series of biopsies from 14 individuals with and without liver cirrhosis (2019). We employed the MVAF value in this dataset to assess its reliability of MVAF in real biological scenarios.

**Fig. 1:**
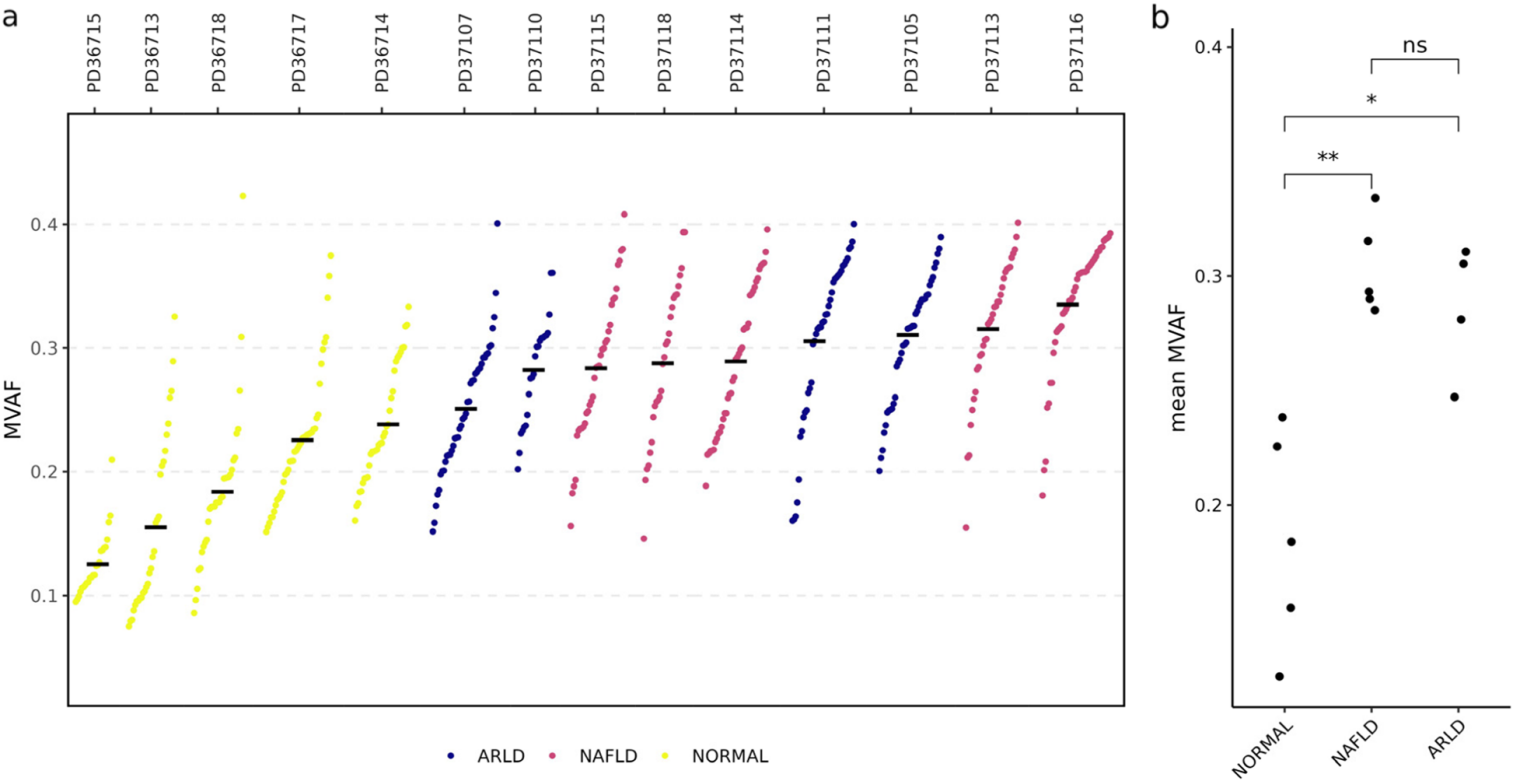
Validation of the performance of Mean Variant Allele Frequency (MVAF) in measuring the degree of clonal expansion. **a**, The MVAF in each biopsy from donors in different liver states. Colored dots represent MVAF in each biopsy sample. Black short lines represent the mean MVAF. The labels in the upper panel represent different individuals. ARLD, alcohol-related liver disease; NAFLD, non-alcoholic fatty liver disease; NORMAL, liver in normal state. **b,** The pairwise comparison of clonal expansion between different states of livers. Significant higher clonal expansion was observed in cirrhotic (ARLD and NAFLD) compared to normal livers, but not within the two cirrhotic groups (Mann Whitney Wilcoxon Test *: *p* < 0*.*1, **: *p* < 0*.*01, ns: not significant)

We found that the MVAF pattern in different livers is distinct (Fig. 1a). Then, we calculated the mean MVAF for all biopsies to measure the degree of clonal expansion in each liver. As expected, individuals with cirrhosis display a higher mean MVAF in comparison to those without cirrhosis (Fig. 1b). This result is reasonable because, in the context of cirrhosis, both chronic liver damage and inflammation provide a conducive environment for clonal expansion, leading to the replacement of healthy liver tissue with scar tissue (Sharma and John 2023; Zhu et al. 2019). Furthermore, we made an intriguing observation regarding the comparable degree of clonal expansion between individuals with non-alcoholic fatty liver disease (NAFLD) and alcohol-related liver disease (ARLD). This phenomenon is of interest because NAFLD and ARLD are distinct liver conditions with different underlying causes and clinical presentations. NAFLD primarily involves the accumulation of fat in the liver and tends to be asymptomatic in its early stages, while ARLD is caused by excessive and chronic alcohol consumption and manifests with varying symptoms (Idalsoaga et al. 2020). Hence, the evaluation of liver cirrhosis progression can exhibit significant variability among individuals.

Altogether, our results substantiate the capability of mean MVAF as a quantitative measure of clonal expansion.

### The variation in the degree of clonal expansion demonstrates consistency among individuals

Throughout a human’s lifespan, clonal expansion of somatic cells could be triggered and facilitated by a range of intrinsic and extrinsic factors, leading to varying degrees of clonal expansion in different organs (Li et al. 2021; Manders, van Boxtel, and Middelkamp 2021). To comprehensively investigate this divergence within the human population, we utilized MVAF measurement on a dataset which is consisting of biopsies obtained from nine organs, including the liver, esophagus, cardia, stomach, bronchus, duodenum, colon, rectum, and pancreas, from five individuals (Supplementary Fig. 1).

**Fig. 2:**
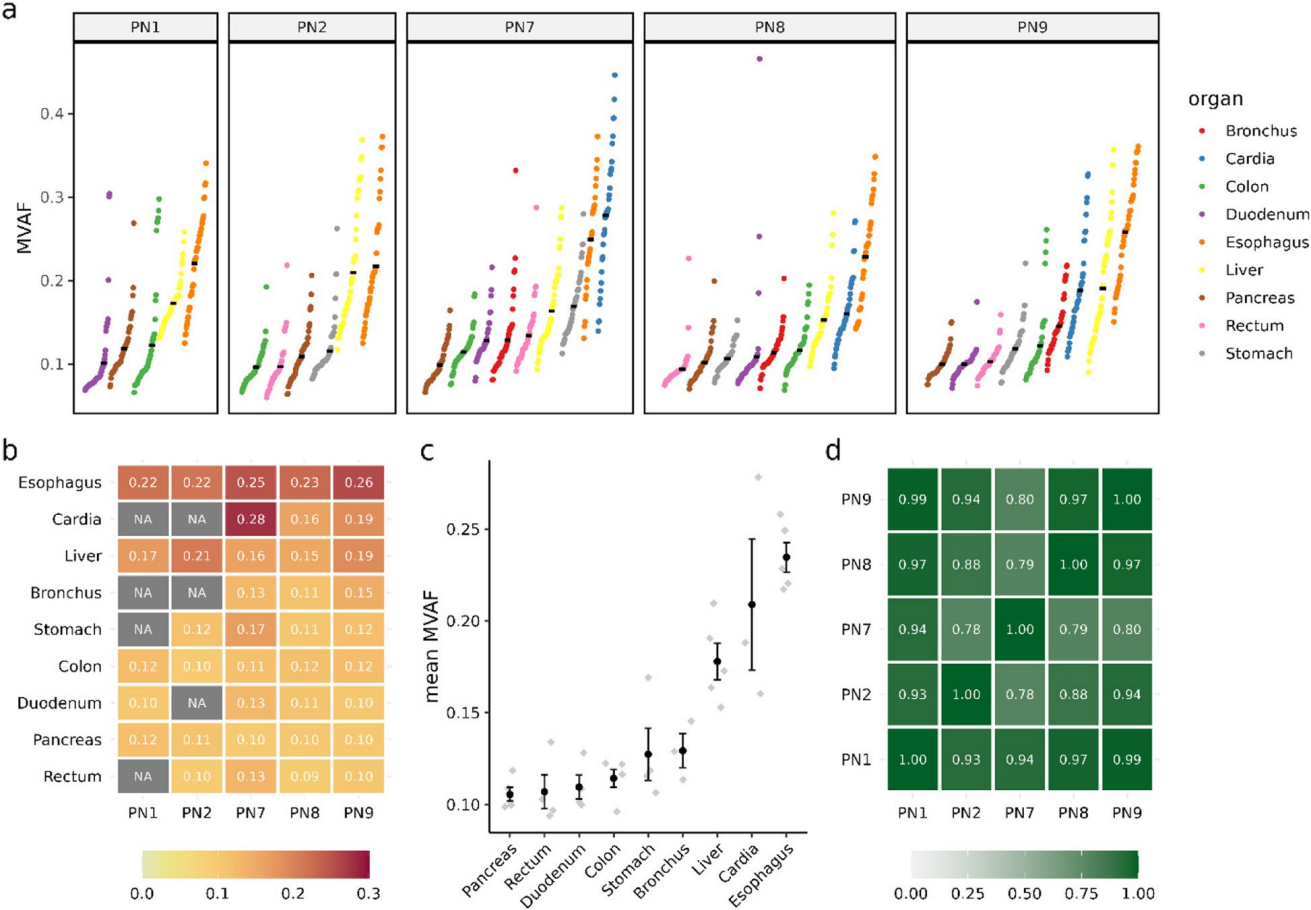
The degree of clonal expansion in nine organs across five individuals. **a**, The MVAF in each biopsy was measured in nine organs from five healthy individuals. Colored dots represent MVAF in each biopsy sample. Black short lines represent the mean MVAF. The labels in the upper panel represent different individuals. **b**, The heatmap for mean MVAF across organs among individuals. **c**, The degree of clonal expansion is distinct across nine organs. The gray dots show the mean MVAF of all biopsies for each organ in each individual. The black dots and error bars indicate the mean and the standard error, respectively. **d**, The variation in the degree of clonal expansion is consistent among organs in different individuals. The heat map illustrates that there is a significant correlation in the mean MVAF across different organs between any two individuals

Our results indicate that there are variations in MVAF among different organs within each individual, and this pattern of variation exhibits a similar trend (Fig. 2a, Fig. 2b). We then measured the degree of clonal expansion for each organ and we found that the esophagus displays clonal expansion signatures at a high level, as previously reported (Martincorena et al. 2018). Interestingly, the liver also exhibits a comparable degree of clonal expansion as the esophagus and cardia (the junction region between the lower esophagus and the stomach). In contrast, the pancreas exhibits the lowest degree of somatic clonal expansion among the nine solid organs (Fig. 2b). To discern the contributions of factors influencing the diversity in mean MVAF, we conducted an analysis of variance (Type II ANOVA). Remarkably, the primary source of variation was attributed to the organ factor, accounting for 83.8% (Supplementary Fig. 2, organ *p* = 1*.*34 × 10^−9^, donor *p* = 0*.*071). It’s noteworthy that the correlation relationship of mean MVAF across all organs is significant for any two individuals, suggesting the variation in the degree of clonal expansion across organs was remarkably consistent within the human population (Fig. 2d). Altogether, our results indicate the robustness and stability of the mean MVAF in measuring the degree of clonal expansion in a population. It should be mentioned that the five individuals examined in this study are all within an age range of 85 to 93, which means that the inferences are not confounded by age, another key factor influencing clonal expansion in somatic cells. In addition, the mean MVAF of the elderly largely reflects the cumulative effects over a human lifespan. This raises an intriguing question about whether organs with a higher degree of clonal expansion are more predisposed to develop tumors.

### The degree of clonal expansion is not correlated with cancer risk

**Fig. 3:**
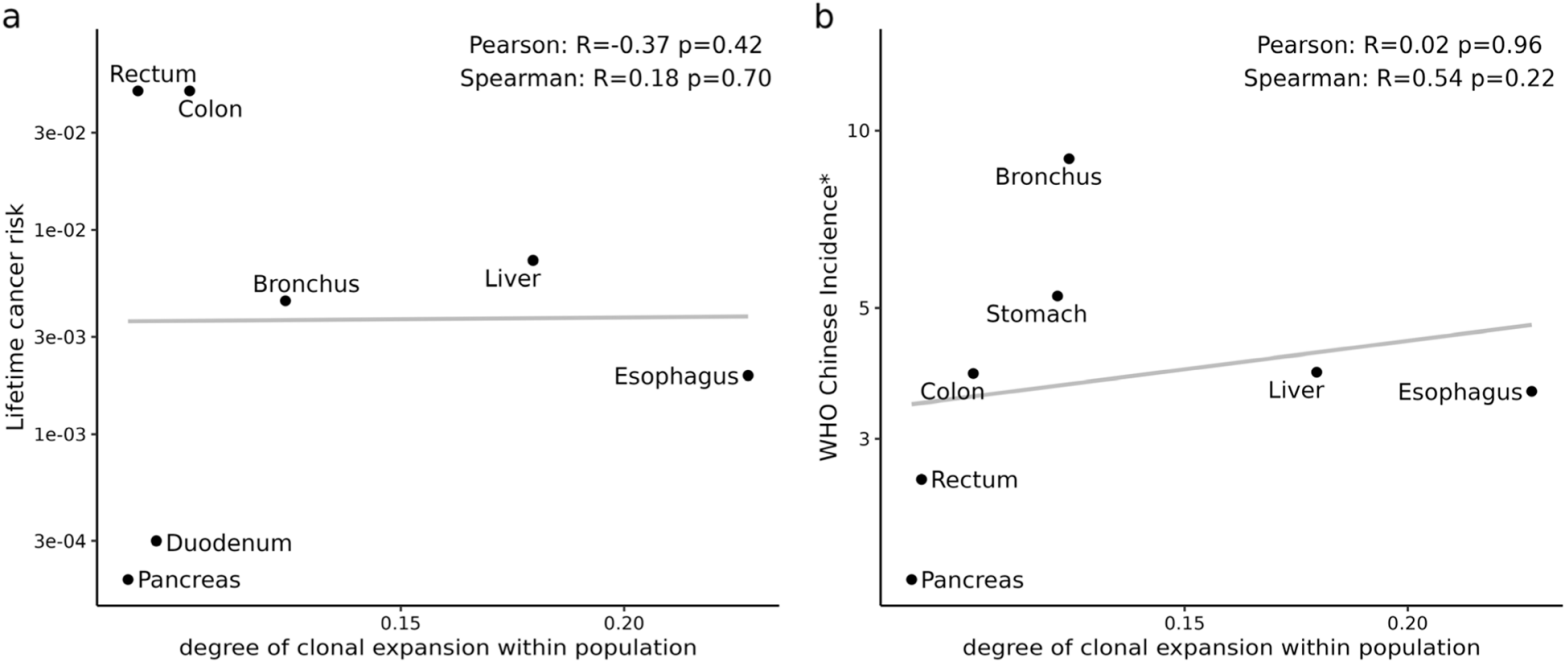
The relationship between the degree of clonal expansion and cancer risk in multiple organs. **a**, **b**, The relationship between the degree of clonal expansion across multiple organs and the corresponding lifetime cancer risk. The data on cancer risk was collected from Tomasetti’s research (2015) (a) and WHO (b), respectively. *: incidence rates per 100,000

To address this issue comprehensively, we conducted a direct correlation analysis between the degree of clonal expansion and the lifetime cancer risk across different organs. On the one hand, we calculated the mean MVAF for each organ among different individuals to measure the degree of clonal expansion in the healthy population. On the other hand, the data of lifetime cancer risk was provided by Tomasetti et al. (2015), who compiled this information from existing literature. Notably, our observations suggest a decoupling of the relationship between the degree of clonal expansion and lifetime cancer risk. (Fig. 3a Pearson *R* =  − 0*.*37, *p* = 0*.*42). For example, both the esophagus and cardia exhibit a higher degree of clonal expansion but have a relatively low lifetime cancer risk. In contrast, colorectal cancer exhibits a higher risk, while the degree of clonal expansion in the elderly population is relatively lower (Supplementary Fig. 3a). Moreover, the number of examined organs is sufficient for correlation analysis, because the correlation relationship between lifetime cancer risk and lifetime stem cell divisions (lscd) remains significant even when considering only seven organs (Supplementary Fig. 3b, Pearson *R* = 0*.*99, *p* = 5*.*05 × 10^−6^).

**Fig. 4:**
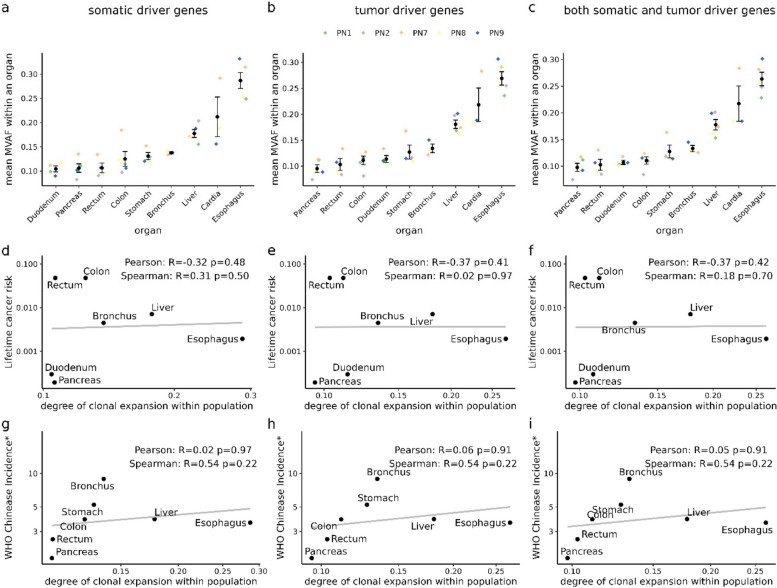
The consistent result of calculating MVAF across SNPs in somatic driver genes, cancer driver genes, or the union of both types of driver genes. **a**, **b**, **c**, The variation in the degree of clonal expansion is consistent across multiple organs using different gene sets. The colored dots show mean MVAF of all biopsies for each organ within each individual. The black dots and error bars indicate the mean and the standard error, respectively. **d**, **e**, **f**, The relationship between the degree of clonal expansion across multiple organs and the corresponding lifetime cancer risk. The data on cancer risk was collected from Tomasetti’s research (2015). **g**, **h**, **i**, The relationship between the degree of clonal expansion across multiple organs and corresponding cancer incidence (WHO). *: incidence rates per 100,000

To eliminate the potential influence of genetic background, we explored an additional cancer statistics dataset specific to the Chinese population, provided by the World Health Organization (WHO). This analysis yields a consistent result (Fig. 3b, Pearson *R* = 0*.*02, *p* = 0*.*96). Additionally, considering that driver genes play a significant role in clonal expansion, we also specifically calculated MVAF across SNPs in somatic driver genes, tumor driver genes, or the union of both types of driver genes, and the results remain consistent (Fig. 4). It implies that the presence of mutations in these specific genes may not directly contribute to an increased risk of cancer. And the results consistently demonstrate stability even after categorizing the SNPs based on their positions and calculating the MVAF across SNPs located within exons, CDS, and UTR (Supplementary Fig. 4). It prompts further investigation into the mechanisms underlying clonal expansion and its relationship with cancer development.

Given that clones carrying early mutations can result in higher MVAF, the use of MVAF as an indicator has limitations. Therefore, we incorporated various metrics to comprehensively evaluate the extent of clonal expansion. For instance, the median VAF separates the higher half from the lower half of the data set, analyzing the quantile 90 VAF helps mitigate the influence of small clones, while the max VAF may indicate the strongest clonal potential capacity. This approach allowed us to capture a more comprehensive view of clonal dynamics. Our findings remained consistent across all measurements, and all the metrics express the similar trends across organs (Supplementary Fig. 5), reinforcing the absence of correlation with cancer risk (Supplementary Fig. 6). In conclusion, our results demonstrate that the degree of clonal expansion in the somatic cell population of solid organs appears to be decoupled from the lifetime cancer risk across organs. This suggests that, although both processes involve a similar accumulation of driver mutations, their underlying mechanisms may differ.

## Discussion

In this study, we utilized mean MVAF as a quantitative measure to evaluate the degree of clonal expansion across human solid organs. At first, we demonstrate the practicability and reliability of mean MVAF in the context of liver cirrhosis. Then we applied this method to assess the divergence of clonal expansion across multiple solid organs among the five elderly individuals, revealing a consistent degree of clonal expansion in the population. This consistency suggests that the divergence of clonal expansion levels among intra-organ samples is much lower than that observed across organs in individuals of similar age. This phenomenon could be attributed to two main factors. On one hand, organs within the same age population typically share basic characteristics, including cell division times and turnover rates of stem cells, leading to similar features of clonal expansion (E. Mitchell et al. 2022). On the other hand, different organs may encounter different environments and undergoing specific biological processes. For example, the esophagus is exposed to external stimuli from food (Martincorena et al. 2018; Yokoyama et al. 2019), the skin is frequently exposed to UV radiation (Jonason et al. 1996; Martincorena et al. 2015) and the relatively higher degree of clonal expansion in liver might result from its strong regeneration capacity due to detoxification processes(Sharma and John 2023). To address the intra-organ divergence across individuals, further research with a larger number of individuals with different disease states, age, and dietary habits is needed. Finally, we delved into the relationship between the clonal expansion of somatic cells and carcinogenesis across the human lifespan. Notably, despite some shared characteristics between these two processes, we found no correlation between somatic clonal expansion and cancer risk across human organs. We should note that similar results were found after filtering out the SNPs occurring in somatic driver genes and cancer driver genes (Fig. 4), and the location of SNPs does not influence the consistency of the results (Supplementary Fig. 4). This intriguing result indicates that somatic clonal expansion and carcinogenesis may involve different molecular mechanisms, following separate evolutionary trajectories within solid organs. For example, several well-known cancer driver genes exhibit higher frequencies of alterations in normal organs compared to cancers, like NOTCH (Abby et al. 2023), ERBB (Lee-Six et al. 2019), and MUC6 (Li et al. 2021). It implies that these driver mutations might provide some advantages to the normal organism (Abby et al. 2023). In addition to the contribution of driver mutations, genome instability, epigenetic modification, and the microenvironment may play distinct roles in two processes.

Considering the disparity identified between hematological and solid tumors in this analysis, we applied an MVAF-based algorithm to the peripheral blood and found a strong clonal expansion in the elder samples, and we found that the MVAF value can effectively distinguish differences in clonal expansion extent across healthy humans in different age groups (Supplementary Fig. 7). However, due to the absence of the peripheral blood in Li’s dataset, it remains challenging to investigate the difference between the peripheral blood and the solid organs directly. Therefore, further investigations should involve a larger sample size, encompassing a wider variety of organs, and a greater number of individuals to enhance the robustness of the results. Taken together, our study provides a novel perspective for examining and comprehending the distinction and potential association between somatic clonal expansion and carcinogenesis.

## Supplementary Information

Supplementary Material 1.

## Data Availability

All data used in this study were obtained from publicly available sources online. The original code to reproduce this research will be deposited in GitHub (https://github.com/Janzulene/No-correlation-between-somatic-clonal-expansion -and-cancer-risk-across-human-organs).
