# Supplementary material for: Variation in cancer risk between organs can not be explained by the degree of somatic clonal expansion
Source: Adv Biotechnol (Singap). 2024 May 23;2(2):18. doi: 10.1007/s44307-024-00025-9 (PMC11740857; doi:10.1007/s44307-024-00025-9)
Supplement: Supplementary file 1 — Supplementary Material 1. [file 44307_2024_25_MOESM1_ESM.docx]

**Supplementary Materials**

**Variation in cancer risk between organs can not be explained by the degree of somatic clonal expansion**

Di Zhang^1^*^,^*^†^, Ao Zhang^1^*^,^*^†^, Xionglei He^1^*^,^*^∗^ & Shanjun Deng^1^*^,^*^∗^

^1^State Key Labratory of Biocontrol, School of Life Sciences, Sun Yat-San University, Guangzhou 510275, China.

^†^These authors contributed equally to this work.

^∗^Correspondence should be addressed to S.D.(dengshj8@mail.sysu.edu.cn); X.H.(hexiongl@mail.sysu.edu.cn).

This PDF file includes:

Supplementary Figures (Supplementary Fig.1-7)

**Supplementary Fig.1:**


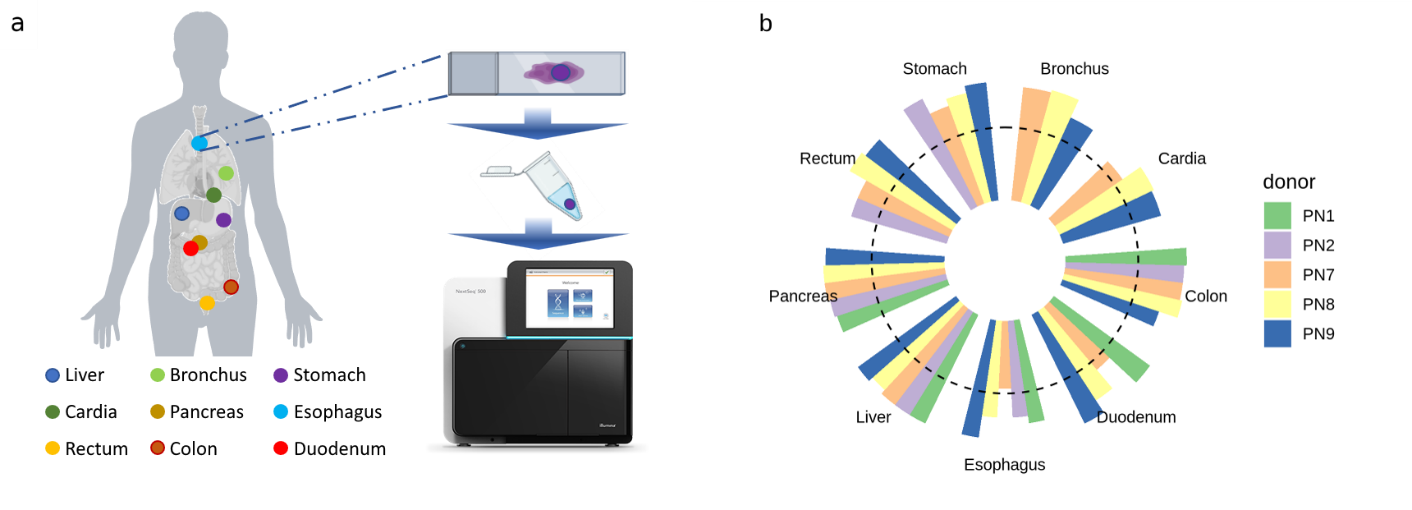


The research strategy for the somatic mutations data in normal organs. a, laser capture microdissection (LCM) and mini-bulk exome sequencing procedure (icons sourced from [http://biorender.com](http://biorender.com/) and website of illumina company). b, The numbers of microbiopsies collected from different organs across five individuals are evenly distributed. The height of each bar represents the number of samples for each organ, and the black dashed line indicates the number of 30, which indicates that all the samples have sufficient biopsies.

**Supplementary Fig.2:**


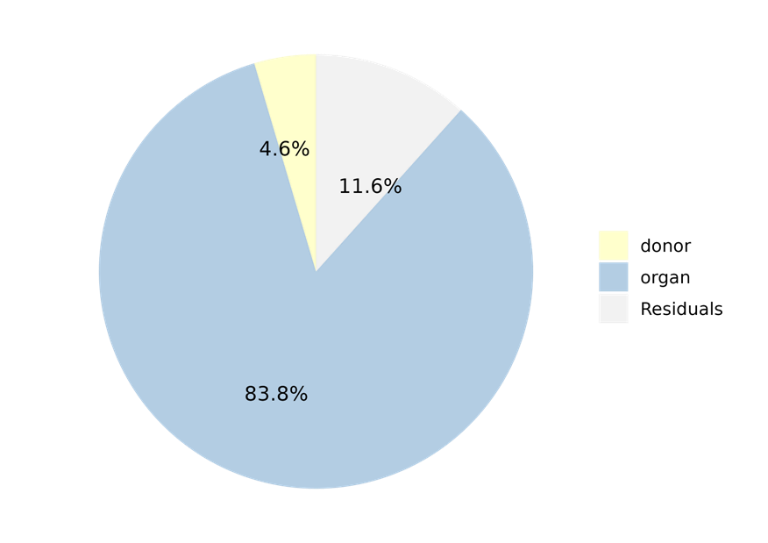


The pie chart represents the proportions contributed by each factor in ANOVA. Different colors represent the factors contributing to the variation in MVAF.

**Supplementary Fig.3:**


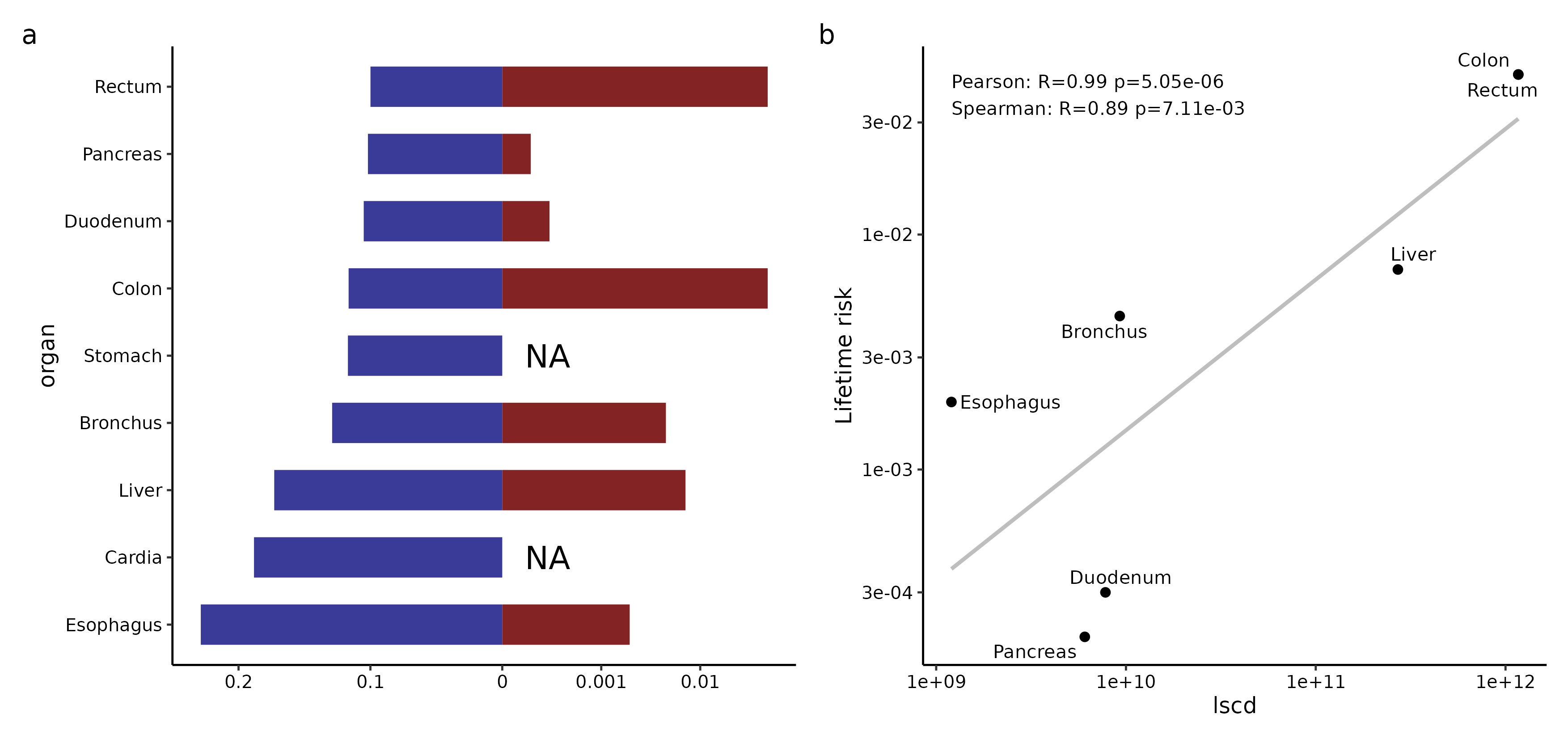


The diagram showing the relationship between clonal expansion and lscd with cancer risk separately. a, Comparison of lifetime cancer risk and the degree of clonal expansion across organs. Blue bars show the degree of clonal expansion sorted in ascending order. Red bars show corresponding lifetime cancer risk for each organ. “NA” denoting unavailable data. b, A strong positive correlation between lifetime stem cell divisions (lscd) and cancer risk for only seven organs, consistent with the study by Tomasetti et al (2015).

**Supplementary Fig.4:**


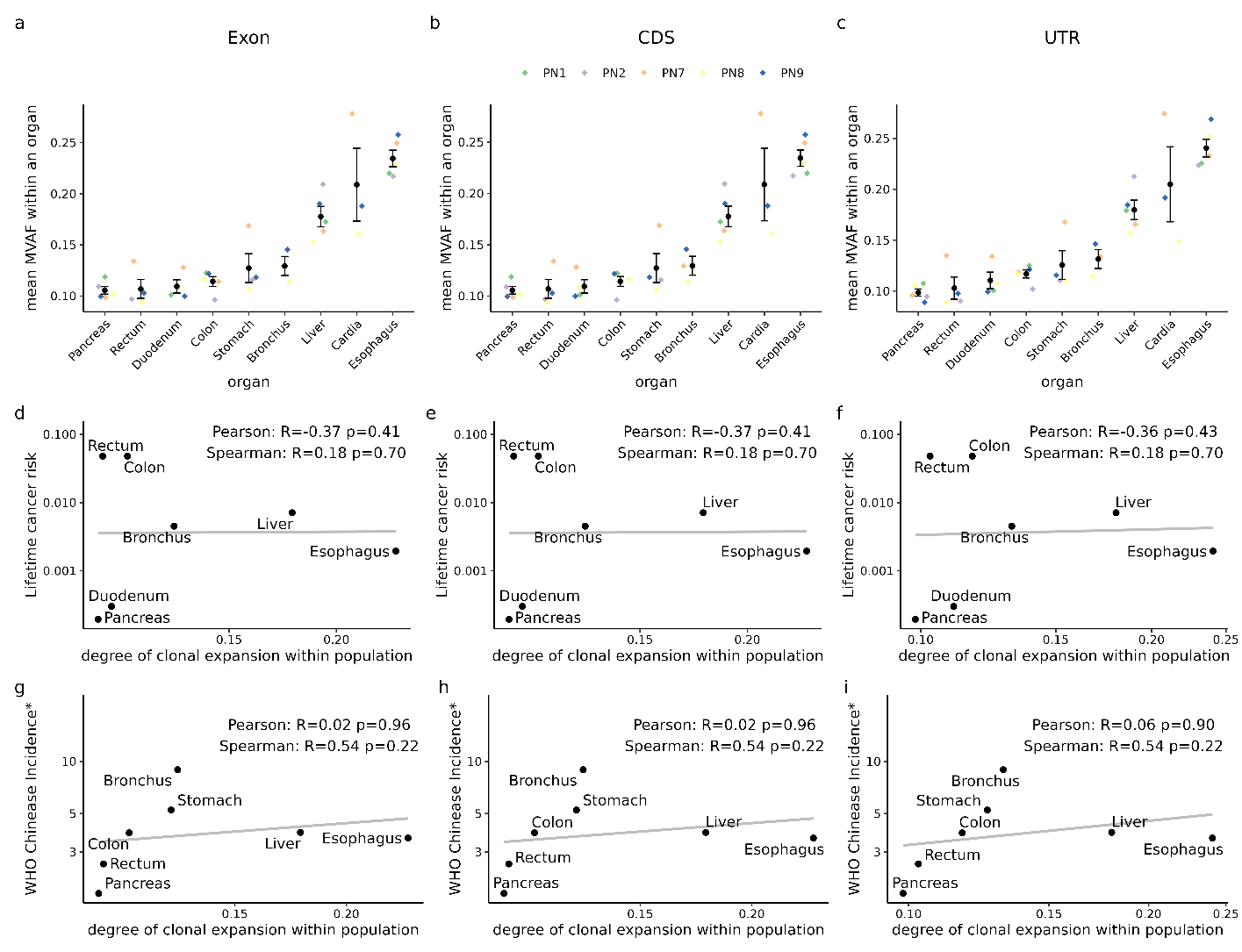


The consistent result of calculating MVAF across SNPs locating in exon, CDS and UTR. a,b,c, The variation in the degree of clonal expansion is consistent across multiple organs when measuring MVAF by different locations. The colored dots show the average value for MVAF across all biopsies for each organ within each individual. The black dots and error bars indicate the mean and the standard error, respectively. d,e,f, The relationship between the degree of clonal expansion across multiple organs and corresponding lifetime cancer risk. The data on cancer risk was collected from Tomasetti’s research(2015)**.** g,h,i, The relationship between the degree of clonal expansion across multiple organs and corresponding cancer incidence (WHO). *: incidence rates per 100,000.

**Supplementary** **Fig.5:**


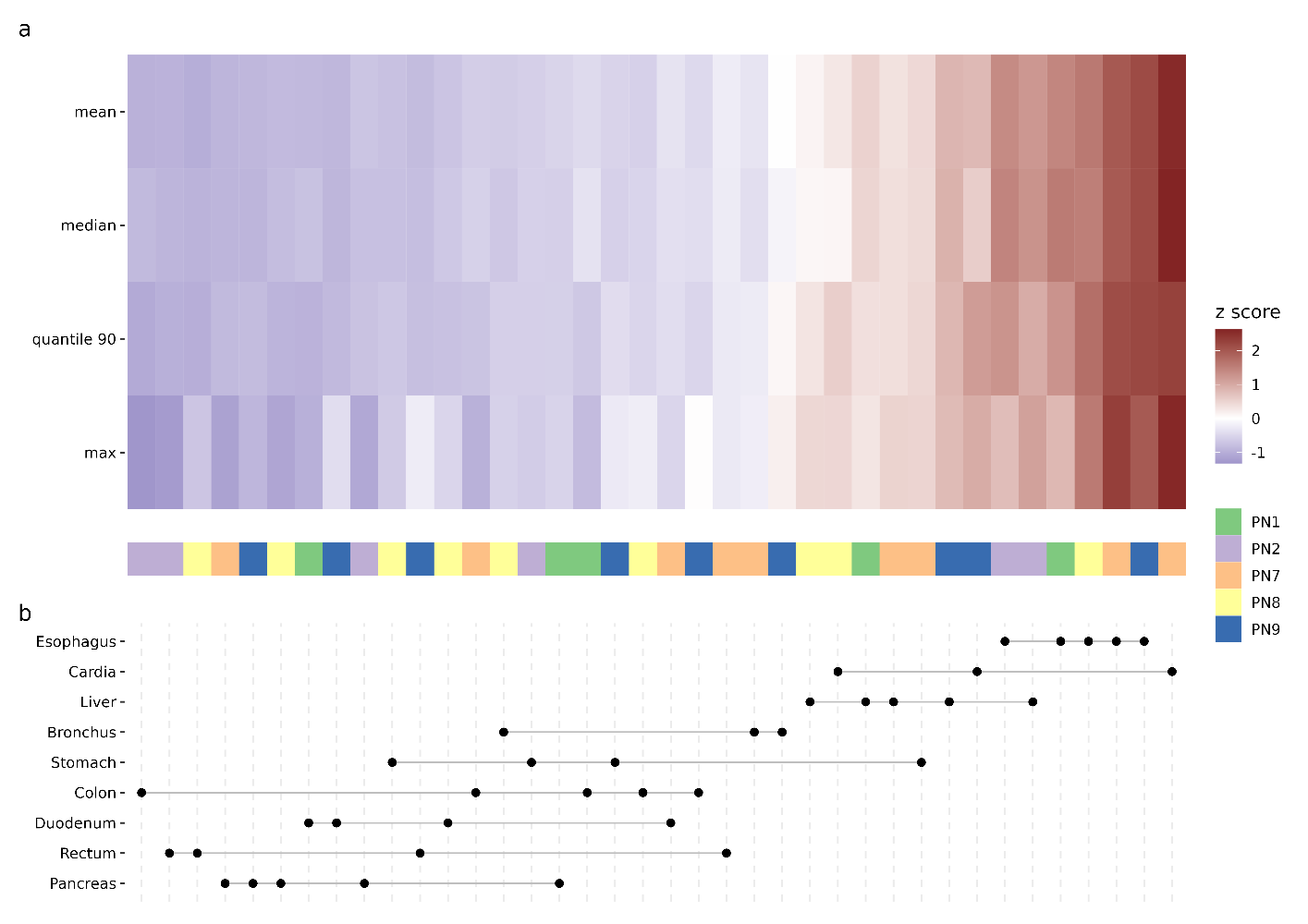


The degree of clonal expansion calculating by different metrics. a, The heatmap for the degree of clonal expansion taken by different metrics, including mean, median, quantile 90 and max of VAF. The value was normalized by z-score, and the colored bars below panel a represent different individuals. b, The rank of clonal expansion degree is consistent among organs in different individuals. The black dots represent the individuals corresponding the colored bars. The lines links to the same organs.

**Supplementary Fig.6:**


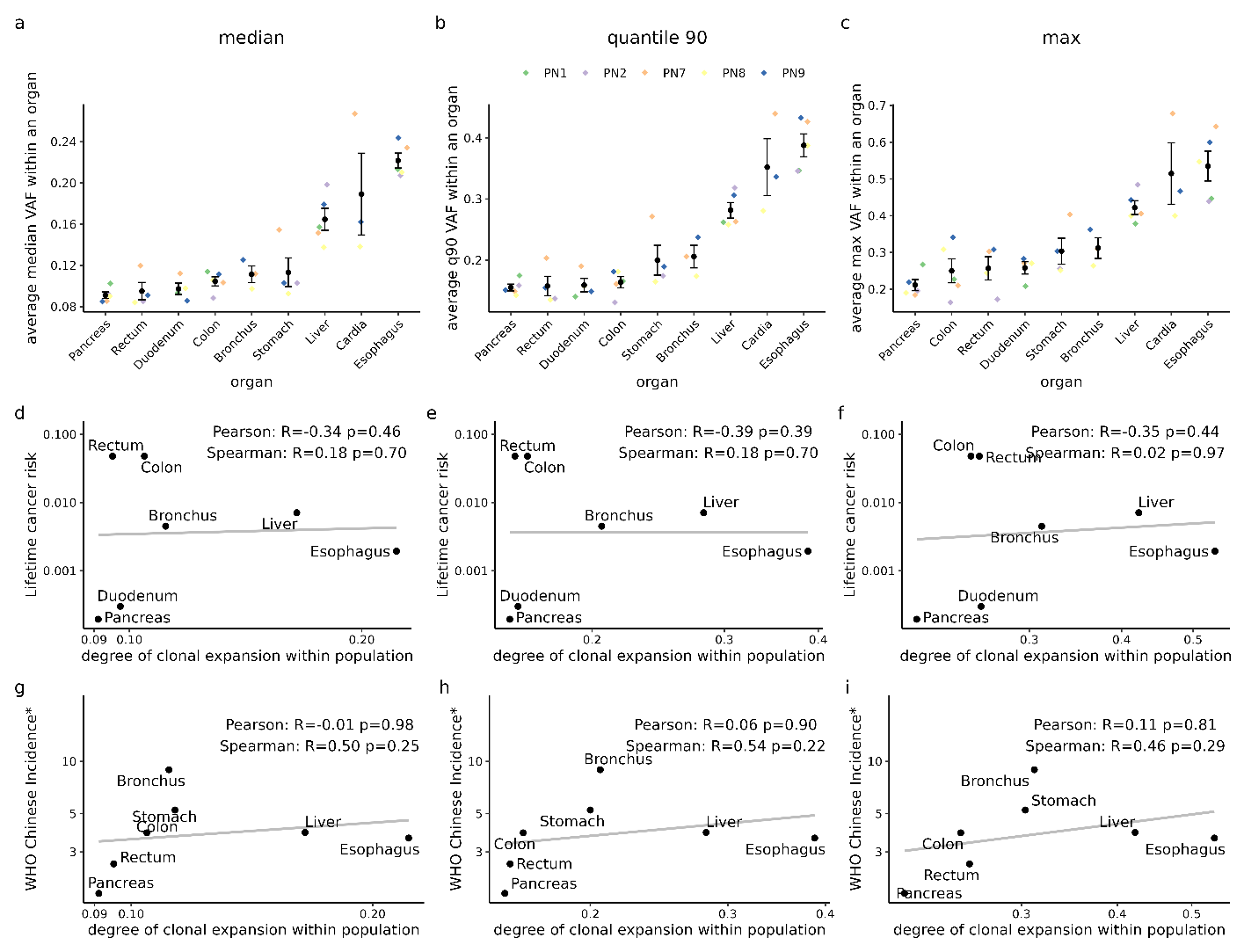


The consistent result of across SNPs using different metrics. a,b,c, The variation in the degree of clonal expansion is consistent across multiple organs measuring by different metrics. The colored dots show average values for the median, quantile 90, max of VAF across all biopsies for each organ within each individual. The black dots and error bars indicate the mean and the standard error, respectively. d,e,f, The relationship between the degree of clonal expansion across multiple organs and corresponding lifetime cancer risk. The data of cancer risk was collected from Tomasetti’s research(2015)**.** g,h,i, The relationship between the degree of clonal expansion across multiple organs and corresponding cancer incidence (WHO). *: incidence rates per 100,000.

**Supplementary Fig.7:**


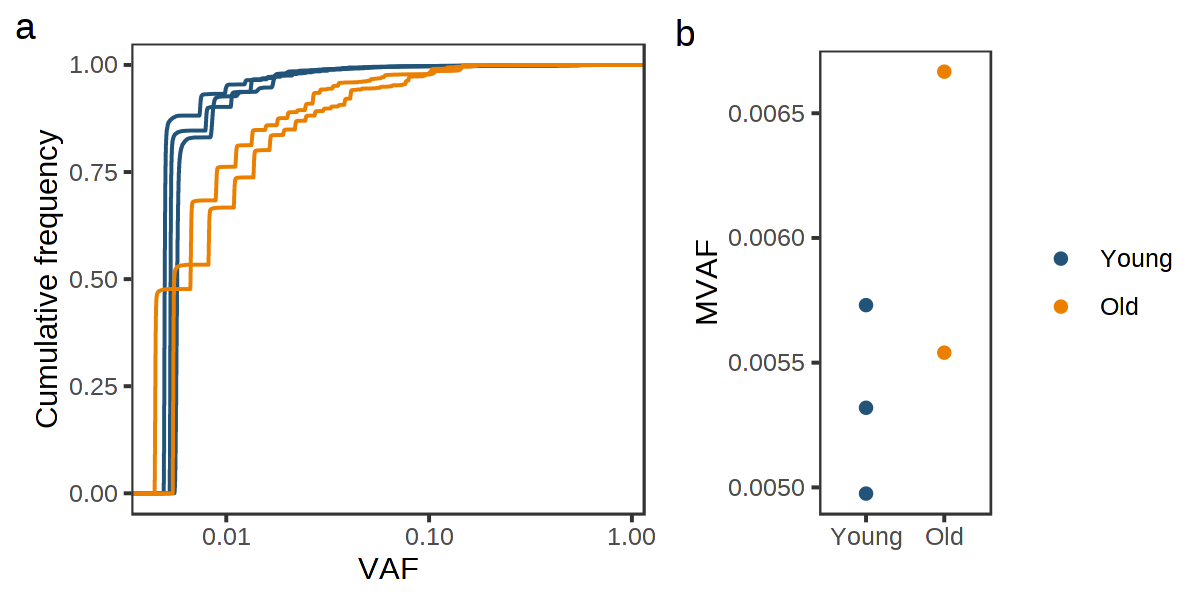


The degree of clonal expansion in healthy human subjects across different ages. a, The curve shows the cumulative frequency of allelic frequency for each individual. The color denotes the age of the individuals, with the young represented in blue and the old in orange. b, The scatter plot displays the MVAF for each individual, with the color representing the age of the individuals as shown in panel a.
